# Identification of Single Nucleotide Polymorphisms Related to the Resistance Against Acute Hepatopancreatic Necrosis Disease in the Pacific White Shrimp *Litopenaeus vannamei* by Target Sequencing Approach

**DOI:** 10.3389/fgene.2019.00700

**Published:** 2019-08-02

**Authors:** Qian Zhang, Yang Yu, Quanchao Wang, Fei Liu, Zheng Luo, Chengsong Zhang, Xiaojun Zhang, Hao Huang, Jianhai Xiang, Fuhua Li

**Affiliations:** ^1^Key Laboratory of Experimental Marine Biology, Institute of Oceanology, Chinese Academy of Sciences, Qingdao, China; ^2^Laboratory for Marine Biology and Biotechnology, Qingdao National Laboratory for Marine Science and Technology, Qingdao, China; ^3^University of Chinese Academy of Sciences, Beijing, China; ^4^Hainan Grand Suntop Ocean Breeding Co., Ltd., Wenchang, China; ^5^Center for Ocean Mega-Science, Chinese Academy of Sciences, Qingdao, China

**Keywords:** single nucleotide polymorphism, target sequencing, association analysis, disease resistance, penaeid shrimp

## Abstract

Acute hepatopancreatic necrosis disease (AHPND) is a major bacterial disease in Pacific white shrimp *Litopenaeus vannamei* farming, which is caused by *Vibrio parahaemolyticus*. AHPND has led to a significant reduction of shrimp output since its outbreak. Selective breeding of disease-resistant broodstock is regarded as a key strategy in solving the disease problem. Understanding the relationship between genetic variance and AHPND resistance is the basis for marker-assisted selection in shrimp. The purpose of this study was to identify single nucleotide polymorphisms (SNPs) associated with the resistance against AHPND in *L. vannamei*. In this work, two independent populations were used for *V. parahaemolyticus* challenge and the resistant or susceptible shrimp were evaluated according to the survival time after *Vibrio* infection. The above two populations were genotyped separately by a SNP panel designed based on the target sequencing platform using a pooling strategy. The SNP panel contained 508 amplicons from DNA fragments distributed evenly along the genome and some immune-related genes of *L. vannamei*. By analyzing the allele frequency in the resistant and susceptible groups, 30 SNPs were found to be significantly associated with the resistance of the shrimp against *V. parahaemolyticus* infection (false discovery rate corrected at *P* < 0.05). Three SNPs were further validated by individual genotyping in all samples of population 1. Our study illustrated that target sequencing and pooling sequencing were effective in identifying the markers associated with economic traits, and the SNPs identified in this study could be used as molecular markers for breeding disease-resistant shrimp.

## Introduction

The Pacific white shrimp *Litopenaeus vannamei*, which naturally distributed along the Pacific coasts of Central and South America, has become the primary cultivated shrimp species into many regions of the world (Lu et al., 2017). Since it was introduced in China into the 1980s, it has become the most dominant aquaculture shrimp species in China (Lu et al., 2016; Wang et al., 2017). In recent years, due to intensive culture and environmental deterioration, infectious diseases caused by viruses and bacteria have led to serious production loss (Wang et al., 2015). Acute hepatopancreatic necrosis disease (AHPND), also called early mortality syndrome, is a devastating disease that usually occurs in 35 days after stocking in cultivation ponds, and the mortality reached as high as 40% to 100% (Joshi et al., 2014; Lun et al., 2018). During disease outbreak, the shrimp showed clinical signs of atrophied hepatopancreas and empty midgut (Nunan et al., 2014). It was reported that *Vibrio parahaemolyticus* carrying the *Photorhabdus* insect-related (Pir*^vp^*) binary toxin was the pathogenic agent for AHPND (Lee et al., 2015).

Many immune-related genes were found to be involved in the defense of shrimp against *V. parahaemolyticus* infection. The Rho signaling pathway was suggested to be helpful for AHPND pathogenesis in shrimp through transcriptome analysis (Ng et al., 2018). Infection of *V. parahaemolyticus* could strongly activate the genes involved in cell growth and anti-apoptosis (Zheng et al., 2018). In addition, the expression of genes encoding immune effectors, including lectins and antimicrobial peptides (AMPs), was significantly up-regulated after *V. parahaemolyticus* challenge (Soonthornchai et al., 2016; Qi et al., 2017; Maralit et al., 2018; Qin et al., 2018). Although investigation on the mechanism of AHPND outbreak in shrimp has been conducted, there is still no effective strategy to control this disease.

Breeding disease-resistant broodstock was regarded as an efficient approach (Moss et al., 2012). Compared to the traditional breeding technique, marker-assisted selection (MAS) is more efficient, time-saving, and free from environmental impact (Lu et al., 2018; Wang et al., 2017). Previous studies indicated that gene polymorphism was closely associated with economic traits (Wang et al., 2016a; Li et al., 2017). Immune-related genes were usually considered as optimal candidates for the selection of markers associated with resistance to pathogens (Guo et al., 2013; Hao et al., 2015). Single nucleotide polymorphisms (SNPs) are a type of widely used markers for MAS due to its high polymorphism and abundance in the genome. It has already been used in aquatic species to facilitate selection breeding and speed up the discovery of genes related to the economic trait, such as high growth (Lv et al., 2015; Tsai et al., 2015), disease resistance (Yue et al., 2012), precocity (Xu et al., 2016), and body conformation (Geng et al., 2017).

Over the past years, molecular genetic studies on the penaeid shrimp *L. vannamei* have made a great progress, such as identification of high-throughput SNPs from the transcriptome (Yu et al., 2014) and construction of high-density linkage map (Yu et al., 2015; Jones et al., 2017). These studies provided fundamental information to identify quantitative trait genes and quantitative trait nucleotides (QTNs) for the AHPND-resistant trait. QTNs have been identified in *L. vannamei* for several traits, such as growth-related trait (Andriantahina et al., 2013), ammonia-tolerant trait (Lu et al., 2018), and WSSV resistance trait (Liu et al., 2014a). Screening for SNPs or genes associated with AHPND is the basic work to reveal the molecular mechanism of shrimp defense for bacteria infection and it will also be of benefit to MAS or gene-assisted selection in shrimp. However, markers related to the AHPND-resistant trait of shrimp are seldom reported until now. In the present study, genetic association analysis using genotyping by target sequencing method and bulked segregant analysis was performed to screen for disease resistance-related SNPs. The bulks were built by selection of extreme samples from populations (Zou et al., 2016). Consequently, several SNPs or genes were identified to be related to the resistance of the shrimp against *V. parahaemolyticus*. The results would provide new insights into the molecular basis of disease defense and accelerate the breeding of shrimp with disease resistance.

## Materials and Methods

### Experimental Shrimp

Healthy shrimp were obtained from Hainan Grand Suntop Ocean Breeding Co., Ltd. (Wenchang, China) and maintained in filtered seawater at a temperature of 26 ± 1°C with continuous aeration. Before the experiment, the shrimp were kept in the aquarium for 3 days to make them acclimate to the laboratory conditions. The shrimp used in this study were the progeny of mating of two breeding lines. The two breeding lines were bred by 7 years of artificial selection, respectively. The progenies produced by the cross of the breeding lines were genetically similar. Although two populations were consisted of multiple families, their genetic backgrounds were very similar.

### *V. parahaemolyticus* Challenge Tests

*V. parahaemolyticus* was isolated from the diseased shrimp. The *Vibrio* strain was cultured in tryptic soy broth with 2.0% sodium chloride liquid media. The cultured bacteria were further checked by positive polymerase chain reaction (PCR) amplification of *PirA* *^vp^* and *PirB* *^vp^* genes (Han et al., 2015). Bacterial titer was counted with a hemocytometer under a light microscope. The infection dose was set at 2.5 × 10^4^ colony-forming units (CFU)/g (body weight). A total of 236 shrimp with a body weight of 3.98 ± 1.9 g and a body length of 6.89 ± 1.35 cm were used as the first population. About 10 μl bacterial suspension in phosphate-buffered saline (PBS) with 1.0 × 10^5^ CFU of *V. parahaemolyticus* was injected into each shrimp at the site between the IV and V abdominal segments. A total of 270 shrimp with a body weight of 5.25 ± 2.9 g and a body length of 7.45 ± 1.56 cm were used as the second population, and each shrimp was injected with 1.3 × 10^5^ CFU of pathogenic bacteria. At the same time, 60 shrimp were set aside as the control group and injected with an equal volume of sterile PBS. The mortality was recorded for 8 days. The dead shrimp were stored at −80°C until DNA extraction. Susceptible and resistant phenotypes were evaluated according to the survival time after challenge (Sawayama et al., 2017). In each population, 60 samples that died at an early time [16 h postinfection (hpi)] after injection of *Vibrio* were taken as the highly susceptible group, and 60 samples that survived from the infection or died at a later time [6 days postinfection (dpi)] were selected as the resistant group.

### Loads of *V. parahaemolyticus in Vivo*

To explore the dynamic change of *V. parahaemolyticus* in shrimp, 150 healthy shrimp with a body weight of 5.15 ± 1.6 g were injected with 2.5 × 10^4^ CFU/g (body weight). To determine the pathogenic bacteria count in surviving shrimp, six shrimp were randomly chosen from the tank at 0, 1, 2, 3, 8, and 12 dpi. Various tissues were collected from each shrimp, including hepatopancreas, gill, and muscle. Three repetitions were used each time, and two shrimp were mixed together in each repetition. Tissues were dissected aseptically and then homogenized in PBS. Serial 10-fold dilutions of the supernatant with PBS were produced, and 100-μl solution was inoculated onto thiosulfate citrate bile salts sucrose (TCBS) agar. Plates were incubated at 30°C for 18 h. Colonies were counted to estimate the number of viable bacteria per pool of shrimp tissue (Sotorodriguez et al., 2015).

### SNP Genotyping Based on Next-Generation Target Sequencing

DNA was extracted from the muscle of shrimp with the Tiangen Plant Genomic DNA Extraction Kit (Tiangen, Beijing, China) according to the manufacturer’s instructions. DNA concentration was assessed using a NanoDrop 2000 spectrophotometer (Thermo Scientific, USA). A SNP genotyping panel based on the AmpliSeq^™^ method was designed. This panel contained 508 amplicons including 60 immune-related genes with 148 amplicons, and the other 360 amplicons were selected with even distribution along the shrimp genome (almost 10 cM per marker along the genome). The primer sequences of the amplicons are displayed in **Supplemental Table S1**.

Each population was divided into two DNA pools from shrimp with susceptibility and two DNA pools with resistance against *V. parahaemolyticus*. Every DNA pool was generated by combining an equal amount of DNA from 30 susceptible or resistant shrimp. These DNA pools were amplified and next-generation sequencing libraries were constructed using DNA Seq Library Preparation Kit for Amplicon Sequencing-Illumina Compatible (Gnomegen Technologies, San Diego, CA, USA). The amplicons were sequenced using Illumina Hiseq 2500. The raw reads were filtered and then mapped to the target sequence using BWA (version 0.7.12; Li, 2011). SNPs were genotyped using the Genome Analysis Toolkit. The read depth of each allele in the pools was extracted from the VCF file and used to estimate the allele frequency in each pool.

### Association Analysis and Candidate Gene Identification

The difference in allele frequency between the susceptible and resistant groups was analyzed using χ^2^ test. The analysis was performed in the combined population (population 1 + population 2) and *P* values were corrected using the false discovery rate (FDR) according to the method described by Benjamini and Hochberg (1995). The threshold of adjusted *P* was set to 0.05 (5% FDR correction). In view of the consistency of favorable alleles in the two populations, SNPs that showed opposite allele frequencies in two populations were filtered out. Statistical tests were performed using the R statistical software (R Core Team, 2018). Screened SNPs were mapped to the genome of *L. vannamei* (Zhang et al., 2019), and the genes around SNPs were examined for candidate genes according to their locations and functions.

### Validation of the Pooling Genotyping Result

To further validate the pooling genotyping result, three SNPs (ALF6-1__22575_510_57, ALF6-1__22575_510__224, and Marker2060_197) were amplified by individual genotyping method in all samples of population 1. Primers were designed based on the flanking sequence of SNPs (**Table 1**). The PCR program was as follows: 1 cycle of denaturation at 94°C for 5 min, 35 cycles of denaturation at 94°C for 30 s, annealing at 58°C for 30 s, and extension at 72°C for 45 s followed by an extension at 72°C for 7 min. The confirmed PCR products were sequenced by Sanger sequencing in Tsingke Biotech (Qingdao, China). The genotype of each sample was determined based on the sequencing chromatograms.

**Table 1:** Primer sequences and corresponding annealing temperature of genes.

Primer name	Primer sequence (5′-3′)	Expected size (bp)	Annealing temperature (°C)
LvALF-F	GGTGACCAGACCTGCTTTGAG	290	58
LvALF-R	CCAGCTAGGATAACCGTAACATG		
Lv2060-F	TCTTACACACATTCTTCCTGGT	190	58
Lv2060-R	AGAAGTCGTTTGGGGAGATTGT		
qLvALF-F	GGTTTGGCTTCTTCCTCGGT	100	56
qLvALF-R	CCAACACCCGCAGCAAAT		
qLvPI3K-F	AATGGTCGTCAAGCAATGTGG	111	56
qLvPI3K-R	TGTCTAATGTGAGCAAGTCTGTCC		
18S-F	TATACGCTAGTGGAGCTGGAA	136	56
18S-R	GGGGAGGTAGTGACGAAAAAT		

### Transcription Analysis on Two Candidate Genes

Based on the annotation of SNPs located in the genes, two candidate genes, including anti-lipopolysaccharide factor (*LvALF6*) and phosphatidylinositol 3-kinase (PI3K) regulatory subunit α isoform X2 (*LvPI3K*), were selected for further analysis. Their expression patterns in the shrimp during *V. parahaemolyticus* infection were analyzed. Healthy shrimp with a body weight of 8.39 ± 2.2 g and a body length of 8.89 ± 0.78 cm were injected with 1 × 10^5^ CFU of *V. parahaemolyticus*. The hepatopancreas, hemocytes, and gill of shrimp were collected separately at 1, 6, 24, 48, and 72 hpi. Nine shrimp were collected at each time point and three individuals were put together as one sample. The detailed steps of total RNA extraction and cDNA synthesis were the same as described previously by Wang et al. (2016b). The expression patterns in different tissues were detected by SYBR Green-based quantitative real-time PCR with the primers shown in **Table 1**. The program was as follows: 95°C for 2 min followed by 40 cycles of 95°C for 15 s, 56°C for 15 s, and 72°C for 20 s, and the melting curve analysis was added to the end of each PCR.

## Results

### AHPND Challenge Test

**Figure 1:**
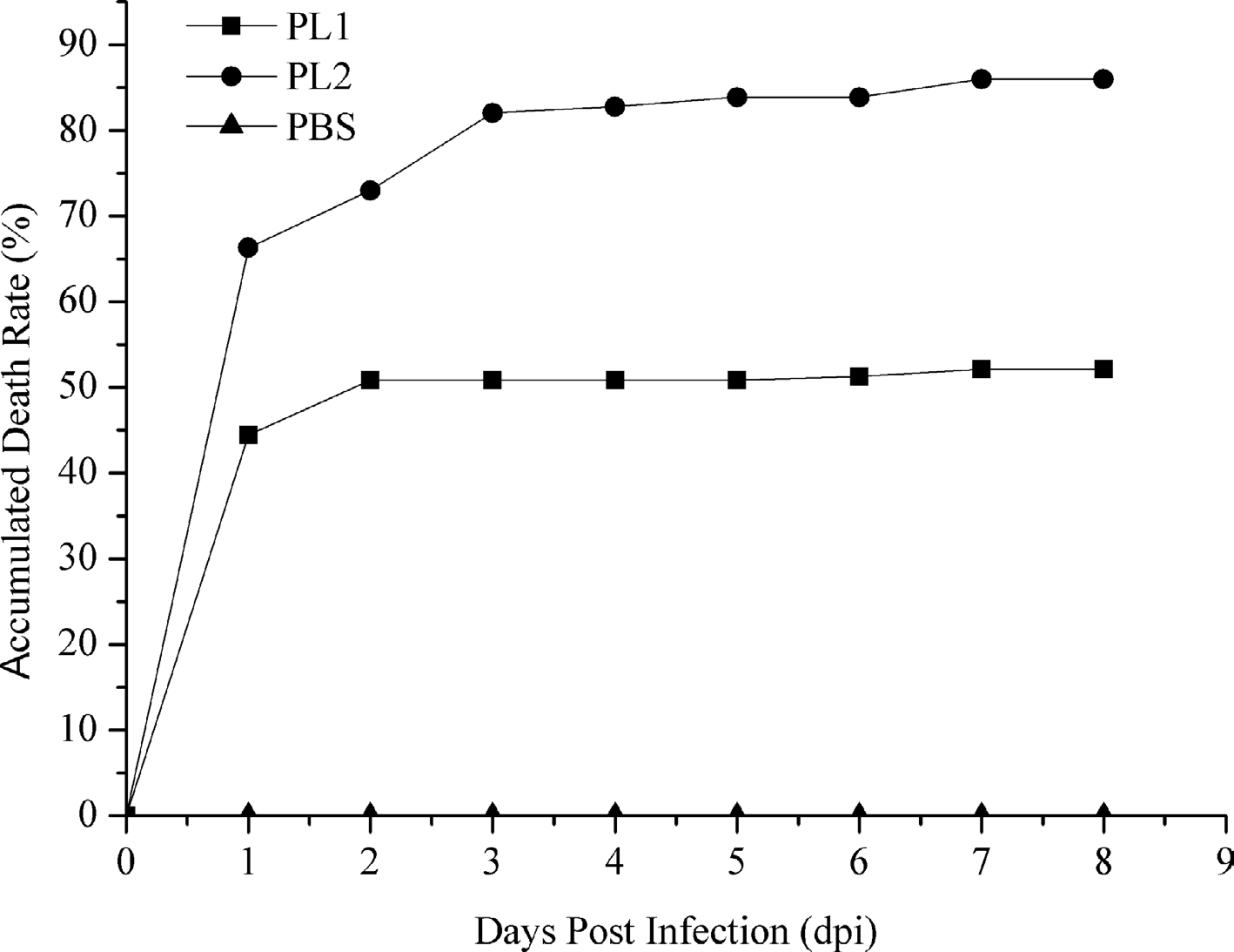
Accumulated mortality rates of populations 1 and 2 infected with *V. parahaemolyticus* and PBS. PL1, population 1; PL2, population 2.

The cumulative mortality of two populations was 52.12% and 86.29%, respectively (**Figure 1**). In population 1, 60 samples that died earlier were selected as the susceptible group, whereas 60 surviving samples were selected as the resistant group. Similarly, 60 shrimp that died first were collected from population 2, whereas 37 surviving shrimp and 23 shrimp that died later were selected as the resistant group. No mortality was observed in the PBS group. The loads of *V. parahaemolyticus* in shrimp at different infection stages are shown in **Table 2**. No *V. parahaemolyticus* was detected in the uninfected shrimp. The number of bacteria in shrimp tissues increased gradually after injection. In hepatopancreas and muscle, the bacterial loads reached 10^6^ and 10^5^ CFU/g at 2 dpi, respectively. The density of bacteria in the gill reached the peak with a bacterial load of 10^3^ CFU/g. At 2 dpi, the bacterial loads decreased slightly. Afterward, it decreased continually in the surviving shrimp and reached 10^2^ to 10^3^ CFU/g in the end.

**Table 2:** Loads of *V. parahaemolyticus* in hepatopancreas, gill, and muscle of shrimp.

dpi	Hepatopancreas (CFU/g)		Gill (CFU/g)		Muscle (CFU/g)	
	Mean	SD	Mean	SD	Mean	SD
0	0	0	0	0	0	0
1	1.49×10^3^	9.80×10^2^	9.73×10^3^	4.06×10^3^	7.78×10^3^	7.46×10^2^
2	1.40×10^6^	1.63×10^6^	3.82×10^3^	2.09×10^3^	2.43×10^5^	1.06×10^5^
3	4.76×10^5^	8.06×10^3^	6.74×10^3^	1.42×10^3^	2.89×10^4^	2.47×10^4^
8	4.82×10^3^	3.95×10^3^	5.25×10^2^	7.50×10	3.11×10^5^	1.80×10^5^
12	1.12×10^3^	2.81×10	5.88×10^2^	1.47×10^2^	6.85×10^2^	1.71×10^2^

### SNPs Associated With AHPND Resistance

**Figure 2:**
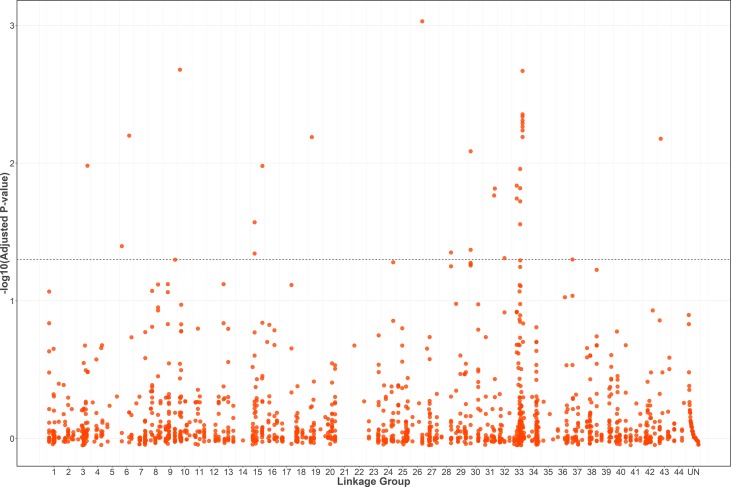
-log_10_(adjusted *P*) of 1556 SNPs (filtering out 10 false-positive sites) in a combined population and the threshold for significance is FDR-corrected *P* < 0.05.

**Figure 3:**
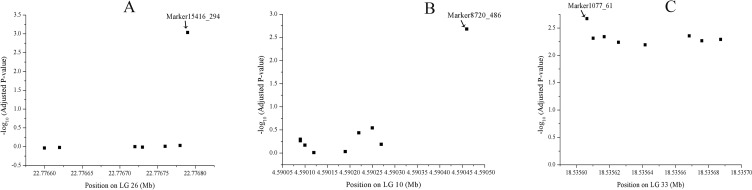
-log_10_(adjusted *P*) of three SNPs, including loci Marker15416_294, Marker8720_486, and Marker1077_61 located in the fragment of Marker15416, **(A)** Marker8720 **(B)**, and Marker1077 **(C)**, respectively.

A total of 1566 SNPs were kept after filtering out SNPs with a minor allele frequency of less than 0.05. The raw data were submitted to the SRA database with accession numbers from SRR9016244 to SRR9016251. Through association analysis on the two populations, 40 significantly different SNPs were identified. After filtering out 10 false-positive sites that showed opposite favorable alleles in two separate populations (**Figure 2**), 30 SNPs were retained as significant markers (**Table 3**). The Manhattan plot of -log_10_(original *P*) and the Q–Q plot were supplied in the supplementary file (**Supplemental Figures S1** and **S2**). Among these 30 markers, 4 SNPs were located in previously selected immune genes and 26 SNPs were located in the fragment distributed along the genome. As several SNPs were located in the same fragment, a total of 17 independent fragments were screened out to be related with the resistance. **Figure 3** shows the -log_10_(adjusted *P*) of three SNPs with the most significant difference, including loci Marker15416_294, Marker8720_486, and Marker1077_61 located in the fragment of Marker15416, Marker8720, and Marker1077, respectively.

**Table 3:** χ^2^ test and allele frequency distribution of SNP markers with significant difference (FDR-corrected *P* < 0.05) in two populations.

SNPs	FDR-corrected *P*	Ref	P1S1	P1S2	P2S1	P2S2	Average	P1R1	P1R2	P2R1	P2R2	Average
Marker15416_294	9.32E-04	A	0.26	0.37	0.47	0.28	0.35	0.18	0.13	0.11	0.21	0.16
Marker8720_486	0.002	A	0.60	0.67	0.61	0.62	0.63	0.50	0.52	0.33	0.36	0.43
Marker1077_61	0.002	T	0.52	0.49	0.57	0.64	0.56	0.43	0.43	0.17	0.38	0.35
Marker1077_126	0.004	C	0.59	0.52	0.59	0.63	0.58	0.44	0.53	0.18	0.41	0.39
Marker1077_72	0.005	C	0.70	0.59	0.67	0.75	0.68	0.51	0.56	0.28	0.58	0.48
Marker1077_65	0.005	T	0.70	0.58	0.67	0.75	0.67	0.51	0.56	0.28	0.58	0.48
Marker1077_146	0.005	T	0.60	0.52	0.60	0.64	0.59	0.47	0.52	0.20	0.43	0.41
Marker1077_134	0.005	C	0.61	0.52	0.59	0.64	0.59	0.46	0.55	0.20	0.42	0.41
Marker1077_81	0.006	C	0.70	0.58	0.67	0.74	0.67	0.51	0.56	0.28	0.58	0.48
Unigene2052_All__14026_558_197	0.006	G	0.84	0.94	0.79	0.80	0.84	0.96	0.97	0.92	0.97	0.96
Marker1077_98	0.006	C	0.60	0.53	0.59	0.64	0.59	0.46	0.54	0.20	0.42	0.40
Marker9677_156	0.006	C	0.44	0.57	0.52	0.50	0.51	0.65	0.60	0.71	0.77	0.68
Marker2060_197	0.007	T	0.71	0.20	0.28	0.54	0.43	0.68	0.77	0.11	0.90	0.61
ALF6-1__22575_510_57	0.008	T	0.39	0.35	0.37	0.58	0.42	0.59	0.55	0.58	0.68	0.60
Unigene19157_All__1806_348_223	0.010	C	0.29	0.44	0.37	0.35	0.36	0.29	0.17	0.17	0.19	0.20
Marker30980_113	0.010	C	0.81	0.82	0.87	0.84	0.84	0.82	0.71	0.55	0.67	0.69
Marker4976_311	0.011	A	0.26	0.39	0.56	0.54	0.44	0.15	0.29	0.27	0.38	0.27
Marker66_168	0.015	A	0.55	0.72	0.47	0.56	0.58	0.72	0.82	0.80	0.62	0.74
Marker4976_524	0.015	T	0.73	0.56	0.52	0.44	0.56	0.88	0.64	0.76	0.64	0.73
Marker17240_299	0.015	C	0.73	0.62	0.65	0.69	0.67	0.31	0.66	0.64	0.41	0.50
Marker8591_231	0.017	C	0.25	0.44	0.48	0.49	0.41	0.31	0.18	0.34	0.16	0.25
Marker66_291	0.018	A	0.42	0.27	0.34	0.18	0.30	0.11	0.24	0.15	0.16	0.16
Marker4976_523	0.019	T	0.73	0.56	0.52	0.47	0.57	0.88	0.65	0.77	0.62	0.73
Marker10592_316	0.027	G	0.89	0.90	1.00	0.97	0.94	0.78	0.75	0.93	0.87	0.83
Marker4976_316	0.028	G	0.26	0.39	0.56	0.56	0.44	0.15	0.33	0.28	0.38	0.28
Marker4311_49	0.040	C	0.01	0.10	0.10	0.11	0.08	0.18	0.06	0.35	0.16	0.19
ALF6-1__22575_510_224	0.043	G	0.69	0.71	0.76	0.86	0.75	0.90	0.84	0.76	1.00	0.88
Marker16977_128	0.045	T	0.92	0.84	0.63	0.77	0.79	0.90	0.95	0.88	0.87	0.90
Marker10592_344	0.045	C	0.91	0.92	1.00	0.97	0.95	0.82	0.78	0.94	0.89	0.86
Marker11943_195	0.049	T	0.39	0.29	0.31	0.44	0.36	0.19	0.13	0.32	0.25	0.22

Ref, allele frequency refers to the allele shown in the “Ref”; P1S1 and P1S2, allele frequency in susceptible bulk of population 1; P2S1 and P2S2, allele frequency in susceptible bulk of population 2; P1R1 and P1R, allele frequency in resistant bulk of population 1; P2R1 and P2R2, allele frequency in resistant bulk of population 2.

To further validate the association result, three SNPs were genotyped by Sanger sequencing in all individuals of population 1. The genotype at the SNP site was determined based on sequencing chromatograms, which is shown in **Supplemental Figure S3**. As for ALF6-1__22575_510_57, 219 samples of population 1 were successfully genotyped. The number of each genotype was 65, 58, and 96 for the A/A, A/T, and T/T genotypes, respectively (**Table 4**). The A/A genotype accounted for 37.5% in the susceptible group, which was higher than that in the resistant group (20.2%). The T/T genotype accounted for 39.2% in the susceptible group, which was lower than that in the resistant group (49.5%). χ^2^ test showed that the shrimp carrying allele T were more resistant than those carrying allele A (*P* = 0.0204). Another SNP in the same gene was ALF6-1__22575_510_224, which also showed significant difference in 219 individuals. The percentage of G in the susceptible group was lower than that in the resistant group (*P* = 0.0293). For Marker2060_197, T was the favorable allele (*P* = 0.031).

**Table 4:** Distribution of the three markers in the susceptible and resistant groups of population 1.

SNP	Genotype	Susceptible, n (%)	Resistant, n (%)	Total, n(%)	**χ** ^2^	P*
ALF6-1__22575_510_57	A/A	45 (37.5)	20 (20.2)	65 (29.7)	7.7839	0.0204
	A/T	28 (23.3)	30 (30.3)	58 (26.5)		
	T/T	47 (39.2)	49 (49.5)	96 (43.8)		
ALF6-1__22575_510_224	A/A	2 (1.68)	3 (3)	5 (2.28)	7.0612	0.0293
	A/G	43 (36.13)	20 (20)	63 (28.77)		
	G/G	74 (62.18)	77 (77)	151 (68.95)		
Marker2060_197	T/T	74 (62.71)	75 (75)	149 (68.35)	6.9488	0.0310
	T/C	10 (8.47)	11 (11)	21 (9.63)		
	C/C	34 (28.81)	14 (14)	48 (22.02)		

*P < 0.05, significant difference of the genotype distributions between these two groups.

### Genomic Regions Associated With AHPND

The corresponding flanking sequences of the above 30 SNPs with significant difference were obtained by blasting the SNP sequence with the assembled reference genome (**Supplemental Table S2**). Among these SNPs, there were only 10 markers located in areas with annotated genes. The surrounding candidate genes are listed in **Table 5**. SNPs ALF6-1__22575_510_57 and ALF6-1__22575_510_224 were both located in *LvALF6*. The SNP Unigene19157_All__1806_348_223 was located in gene *LvPI3K*. In addition, Marker66_168 and Marker66_291 were near the ubiquitin carboxyl-terminal hydrolase. Some other genes, such as low molecular weight phosphotyrosine protein phosphatase-like and calpain-B-like, were also found as candidate genes.

**Table 5:** Information of associated regions and candidate gene identification.

SNPs	LG*	Gene Annotation
Unigene2052_All__14026_558_197	LG6	Low molecular weight phosphotyrosine protein phosphatase-like
ALF6-1__22575_510_57	LG30	Anti-lipopolysaccharide factor
Unigene19157_All__1806_348_223	LG3	PI3K regulatory subunit α isoform X2
Marker30980_113	LG15	Calpain-B-like
Marker66_168	LG33	Ubiquitin carboxyl-terminal hydrolase
Marker17240_299	LG31	Forkhead domain-containing protein crocodile-like
Marker66_291	LG33	Ubiquitin carboxyl-terminal hydrolase
Marker4311_49	LG6	*Penaeus vannamei* protein cornichon homolog 4-like
ALF6-1__22575_510_224	LG30	Anti-lipopolysaccharide factor
Marker11943_195	LG32	TOM1-like protein 2

*LG, linkage group.

**Figure 4:**
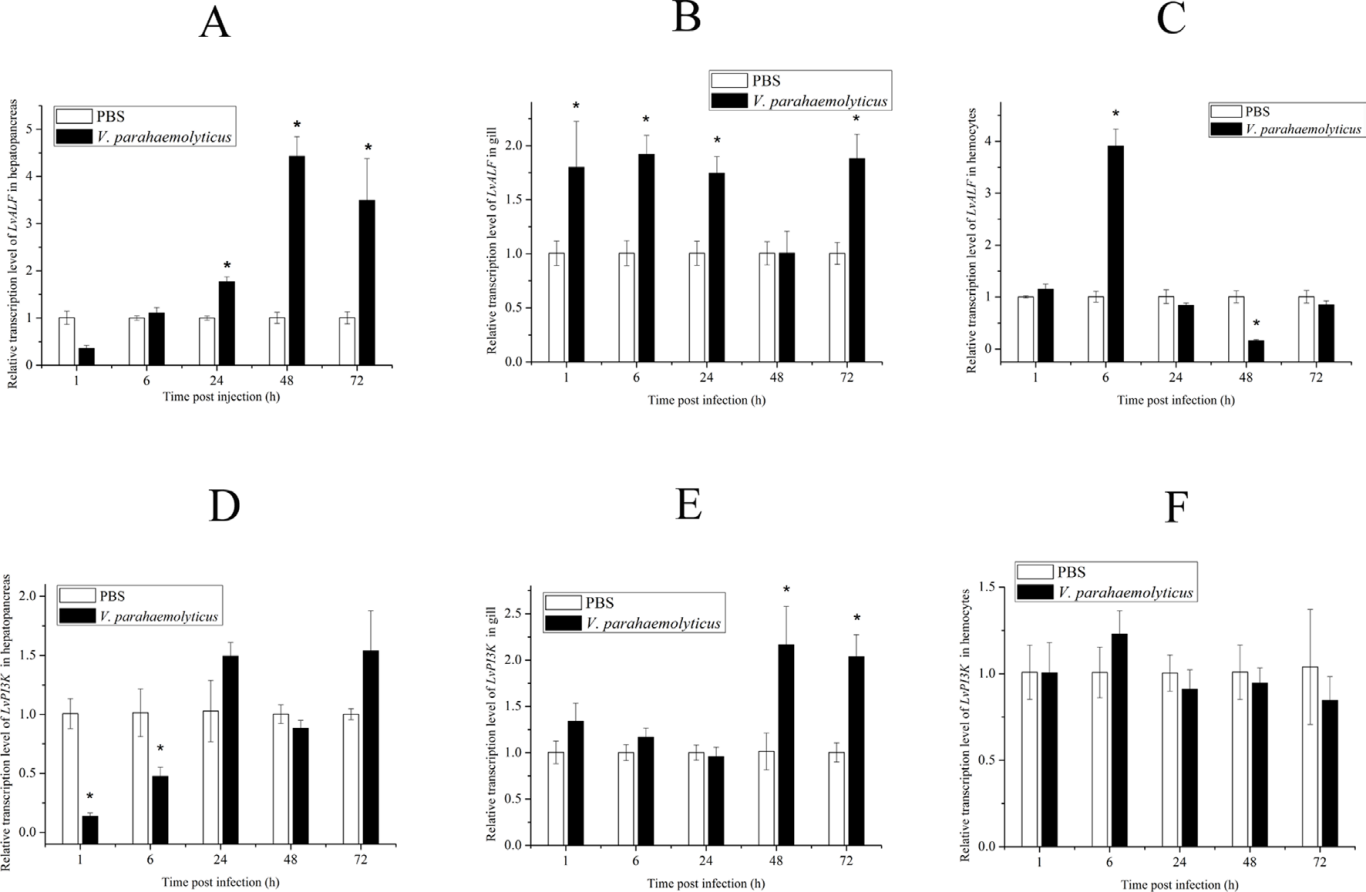
Expression profile of *LvALF6* and *LvPI3K* regulatory subunit in hepatopancreas, gill, and hemocytes at different times after *V. parahaemolyticus* infection (*P* < 0.01). Relative transcription level of LvALF in hepatopancreas **(A)**, gill **(B)** and hemocytes **(C)**; Relative transcription level of LvPI3K in hepatopancreas **(D)**, gill **(E)** and hemocytes **(F)**.

According to the gene annotation and association *P* value, we focused on two candidate genes. One was *LvALF6*, where two SNPs with significant difference were located. The other was *LvPI3K*, which contained one SNP with significant difference designated as Unigene19157_All__1806_348_223 (**Table 3**). For SNP Unigene19157_All__1806_348_223, the average frequency of C allele was 36% in the susceptible group and 20% in the resistant group. Thus, C allele was identified as a deleterious allele for disease resistance. We further analyzed the expression profiles of the two candidate genes after *Vibrio* challenge (**Figure 4**). The expression level of *LvALF6* in hepatopancreas and gill was significantly up-regulated after infection (**Figure 4A, B**), and it was up-regulated at 6 hpi and then down-regulated at the late infection stage in hemocytes (**Figure 4C**). The expression level of *LvPI3K* regulatory subunit in hepatopancreas was significantly down-regulated at early infection stage (**Figure 4D**), whereas it was obviously up-regulated in gill at 48 and 72 hpi (**Figure 4E**). *LvPI3K* showed no expression difference in hemocytes after infection (**Figure 4F**).

## Discussion

In recent years, AHPND has been considered as one of the main problems that hinders the rapid development of the shrimp aquaculture industry. However, no effective method has been reported to control this disease until now. It is widely believed that the selection for a disease-resistant host is one of the valid control measures to prevent disease outbreak in shrimp farming (Cock et al., 2009). A good example is selective breeding of Taura syndrome virus-resistant lines in *L. vannamei* (Argue et al., 2002; Moss et al., 2012). Recently, Ge et al. (2018) reported a three-generation selective breeding of ridgetail prawn (*Exopalaemon carinicauda*) to improve the resistance of AHPND infection. However, no research about the selection of AHPND-resistant broodstock was reported for *L. vannamei* until now. Currently, MAS was known as a method that can greatly accelerate the process of breeding. In red sea bream, it suggested that the RSIVD-resistant trait was controlled by one major quantitative trait loci and could be useful for MAS (Sawayama et al., 2017). Seven SNP markers were identified as markers for the selection of *V. parahaemolyticus* infection resistance in clam (*Meretrix meretrix*; Nie et al., 2015). Compared to other species, there are few studies about molecular markers associated with AHPND in shrimp. Thus, screening of SNP markers associated with disease resistance is the first step to MAS.

In this study, we carried out the *V. parahaemolyticus* challenge experiment to obtain AHPND-resistant and AHPND-susceptible populations. Most of the mortality occurred within the first 48 hpi, whereas there was no mortality in the control shrimp. By investigating the dynamic change of pathogenic bacteria *in vivo*, healthy shrimp were confirmed to be *V. parahaemolyticus* free. The amount of *V. parahaemolyticus* in infected shrimp was higher than that in surviving shrimp, and *V. parahaemolyticus* density was rapidly increased at 1 or 2 dpi, which was in accordance with the highest mortality rate during this time. In the terminal stage of the disease, a decrease of *V. parahaemolyticus* counts was observed due to the immune system mechanisms (Khimmakthong and Sukkarun, 2017).

The approaches of discovering SNPs include whole genome sequencing, large-scale amplicon sequencing, transcriptome sequencing, gene-enriched genome sequencing, and so on (Henry et al., 2012). Here, SNP screening and genotyping were performed using a targeting sequencing panel. It contained genome-wide distributed fragments and immune-related genes, which include immune effective factors or immune signaling pathways such as the JAK/STAT, Toll, and IMD pathways. The target sequencing approach based on multiplex PCR is an efficient approach to analyze genetic variation in specific genomic regions. It is cost-effective, flexible, high throughput, and suitable for non-model species. We applied this method for genotyping genomic regions of interest using pooling genotyping strategy in *L. vannamei*. Subsequently, individual genotyping result proved that pooling sequencing using the designed amplicon panel displayed high sensitivity in detecting allele frequency.

To reduce false-positives, two populations were designed to provide validation for each other. By comparing the allele frequency between the two populations, some SNPs that showed opposite favorable alleles in two separate populations were removed. Among 30 identified associated markers, 14 SNPs were located in LG33. Totally, eight, four, and two SNPs with significant difference were found in the fragment of Marker1077, Marker4976, and Marker66, respectively. We speculate that most of these markers were closely linked. However, these regions have no complete genome annotation. It may result from two reasons: one is that these markers might be closely linked to a candidate gene nearby and the other is that the genome assembly might not be complete; therefore, no candidate genes were identified in this region. With the improvement of the genome assembly, these genomic regions deserve further analysis. Totally, 10 markers were located in the gene region. Several SNPs showing the most significant difference, including loci Marker15416_294, Marker8720_486, and Marker1077_61, were not annotated yet. However, this does not affect the usefulness of SNPs as molecular markers for breeding disease-resistant broodstock.

Among these annotated genes, ALF is a kind of AMP with broad-spectrum activities against bacterial pathogens. A number of ALFs were identified and characterized in *E. carinicauda* (Lv et al., 2017) and Chinese shrimp *Fenneropenaeus chinensis* (Li et al., 2015). Different ALFs exhibited diverse antibacterial and antiviral activities in *E. carinicauda* (Lv et al., 2018) and *F. chinensis* (Yang et al., 2015). In our previous studies, mutations in *LvALF* gene have been regarded as the important immune factor in *L. vannamei* with WSSV resistance (Liu et al., 2014a; Liu et al., 2014b). Several polymorphisms of *PtALF* in the swimming crab (*Portunus trituberculatus*) were reported to be associated with resistance and susceptibility to *Vibrio alginolyticus* (Li et al., 2013). In this study, the expression level of *LvALF6* was obviously up-regulated in the shrimp infected by bacteria compared to those in the control group. Therefore, data suggested that *LvALF6* played an important role in the disease resistance of shrimp.

In addition, SNPs in *LvPI3K* were also identified to be associated with *V. parahaemolyticus* resistance. The PI3K-Akt pathway is an important intracellular signaling pathway involved in various cellular functions, including anti-apoptosis, protein synthesis, glucose metabolism, and cell cycling (Ruan et al., 2014). Several key molecules in this pathway have been reported to be associated with the invasion of some viruses in shrimp (Su et al., 2014; Zhang et al., 2016). Previous studies have reported that *V. alginolyticus* challenge induced an up-regulation of *LvPI3K* expression (Kong et al., 2018), which illustrated that *LvPI3K* might play an important role in *Vibrio* infection.

In summary, we analyzed 1566 SNPs distributed along genome and candidate genes and identified 30 SNPs associated with shrimp resistance to AHPND. The results proved that the target sequencing approach was a useful method for genotyping interested genomic regions and the pooling genotyping method was more cost-effective than sequencing each individual separately, whereas the results were accurate and convinced. The method established in this study and the identified SNPs could be used for the selective breeding of AHPND-resistant broodstock in *L. vannamei*. The candidate disease-resistant genes will provide information in dissecting the resistance response of *L. vannamei* against *V. parahaemolyticus.*

## Data Availability

All datasets generated for this study are included in the manuscript and the supplementary files.

## Author Contributions

QZ and YY conducted the experiment and data processing. JX and FHL conceived and supervised the project. QW contributed to statistical analysis. HH and CZ prepared and cultured the experimental animals. ZL participated in the extraction of genomic DNA. FL contributed to prepare *V. parahaemolyticus*. QZ, YY, and FHL prepared the manuscript. All authors have read and approved the manuscript.

## Funding

This work was supported by the National Natural Science Foundation of China (31830100), the National Key R&D Program of China (2018YFD0901301 and 2018YFD0900103), and China Agriculture Research System-48.

## Conflict of Interest Statement

Author HH was employed by company Hainan Grand Suntop Ocean Breeding Co.,Ltd.Wenchang, China. The remaining authors declare that the research was conducted in the absence of any commercial or financial relationships that could be construed as a potential conflict of interest.

## Supplementary Material

Supplemental Figure S1 Manhattan plot of -log_10_(original *P*).

Supplemental Figure S2 Q-Q plot for *P* values.

Supplemental Figure S3 Chromatograms of different individuals with different allelic variants at the SNP loci (arrows).
