# Supplementary figures and images for: Identification of Single Nucleotide Polymorphisms Related to the Resistance Against Acute Hepatopancreatic Necrosis Disease in the Pacific White Shrimp Litopenaeus vannamei by Target Sequencing Approach
Source: Front Genet. 2019 Aug 2;10:700. doi: 10.3389/fgene.2019.00700 (PMC6688095; doi:10.3389/fgene.2019.00700)

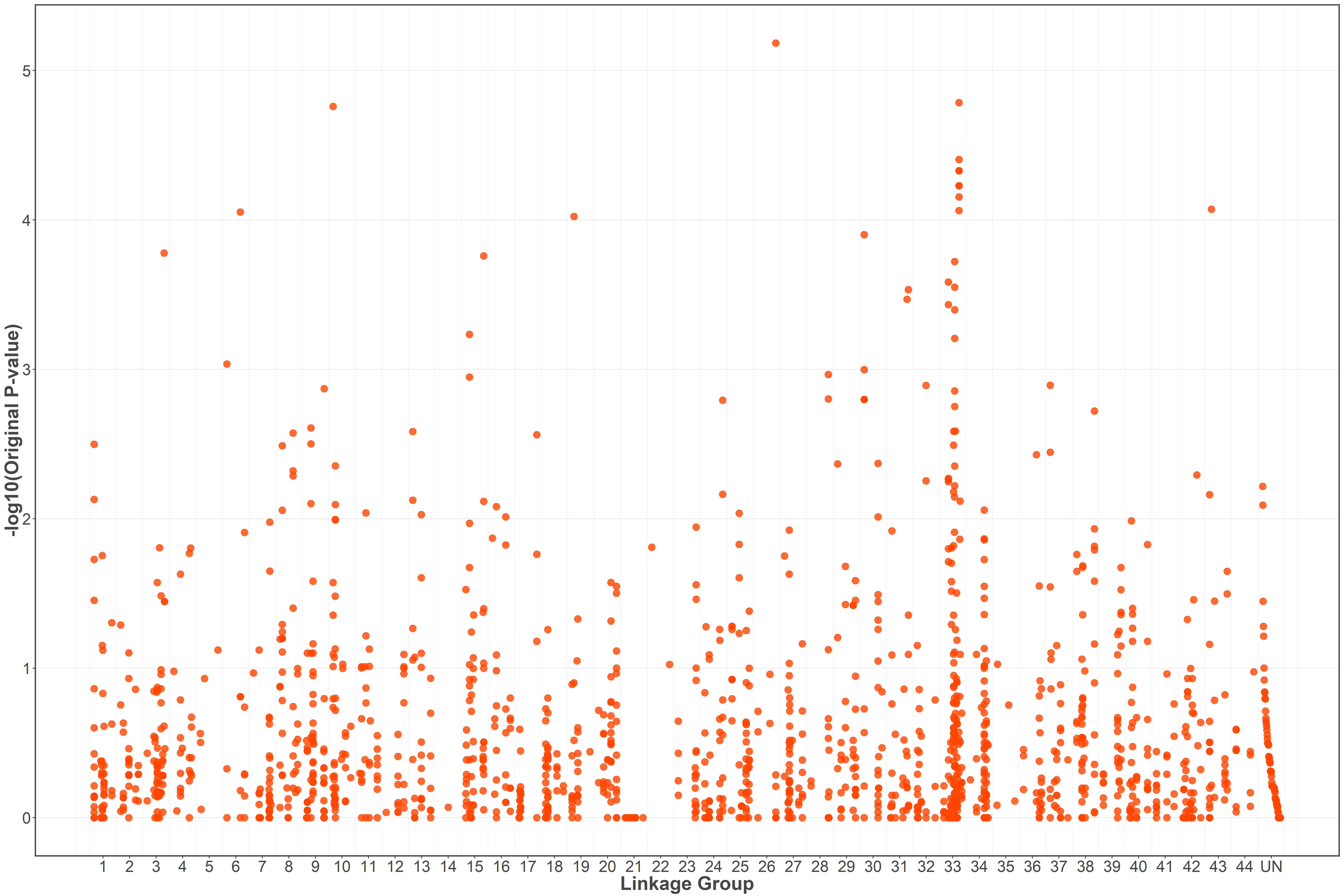

Supplement: Supplemental Figure S1 — Manhattan plot of -log10(original P). [file Image_1.tiff]

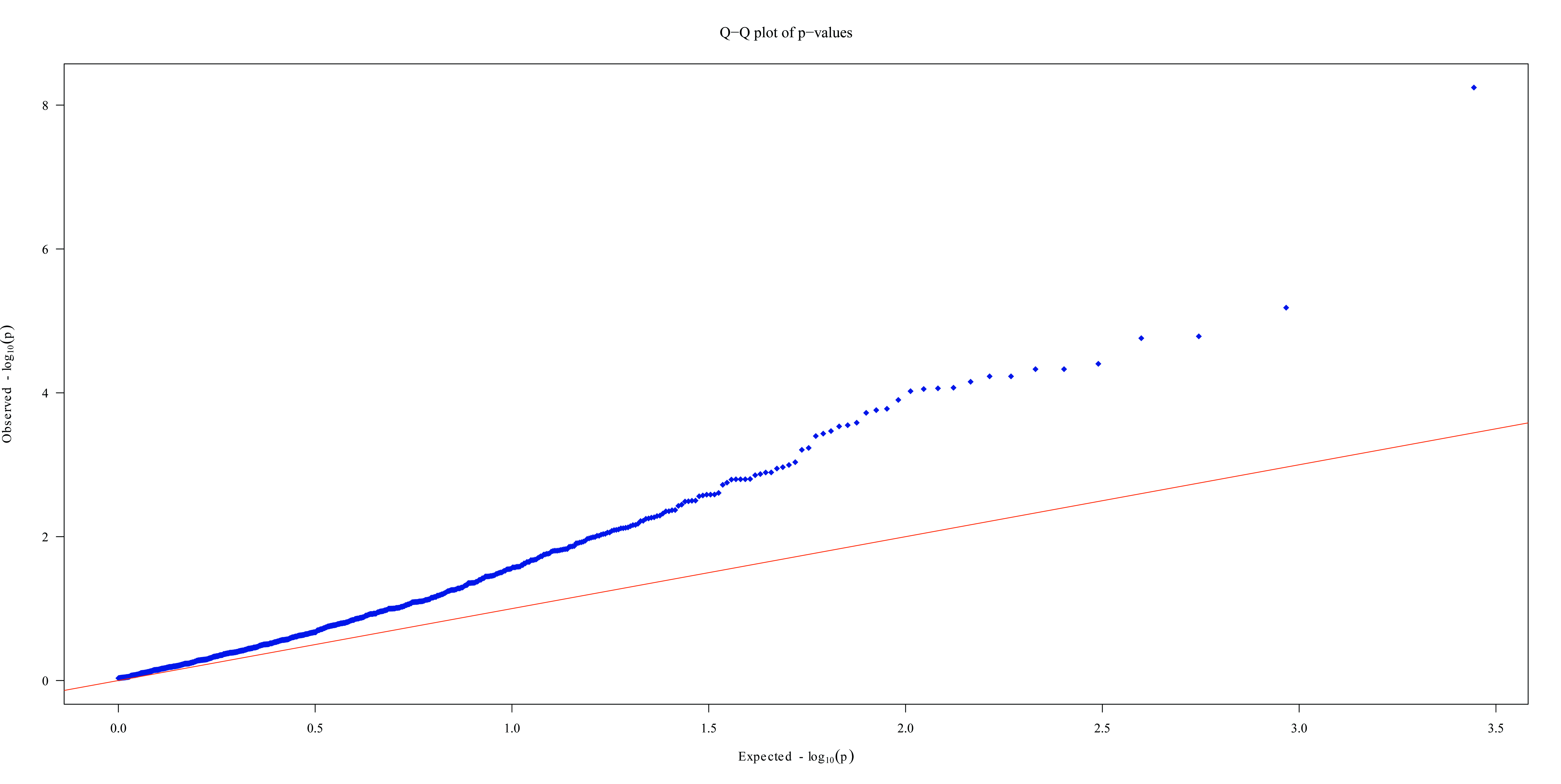

Supplement: Supplemental Figure S2 — Q-Q plot for P values. [file Image_2.tif]

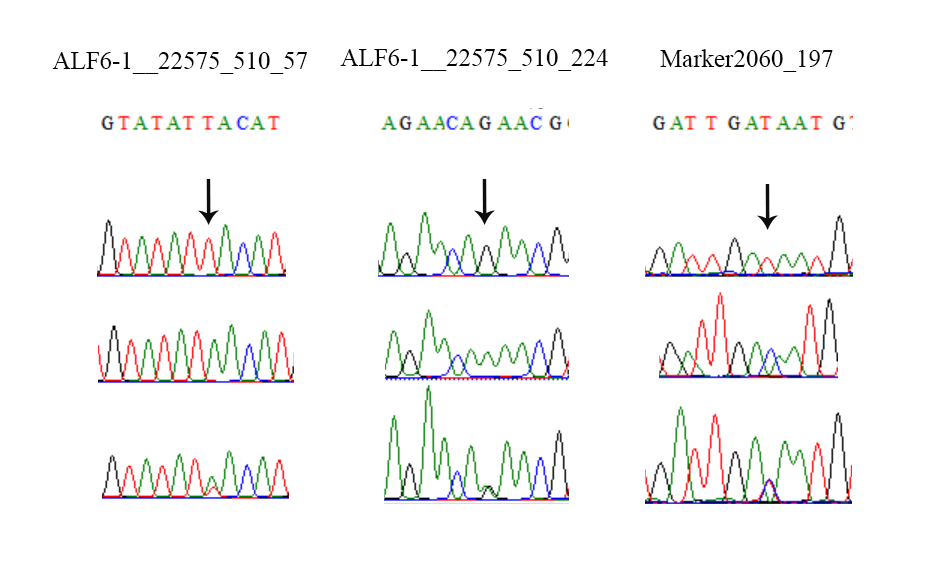

Supplement: Supplemental Figure S3 — Chromatograms of different individuals with different allelic variants at the SNP loci (arrows). [file Image_3.tif]
