# Supplementary material for: Identification of Single Nucleotide Polymorphisms Related to the Resistance Against Acute Hepatopancreatic Necrosis Disease in the Pacific White Shrimp Litopenaeus vannamei by Target Sequencing Approach
Source: Front Genet. 2019 Aug 2;10:700. doi: 10.3389/fgene.2019.00700 (PMC6688095; doi:10.3389/fgene.2019.00700)
Supplement: Supplementary file 5 [file Table_2.docx]

**Supplemental TABLE S2|** The genomic sequences around the 30 SNPs.

| **SNPs** | **Sequence (5' to 3')** |
| --- | --- |
| Marker15416_294 | ATCAAAGTTTTAGTTTTATCTCTTCCTTTTTTACTGCTTTATTCTGATTT**[A/C]**TATTTCCATTCTCTTTGATTTCTCTTGCTCCCCCTTCTCCCTCTCCTTTT |
| Marker8720_486 | GCCGAGAGTTCCCCTGTTTTATGTCGCGCTGTTGGCCTCCTTTTCCACGT**[A/C]**CGGTTAGGTTAAGGTTAGGTTAGGTGTTAGGTTTTAAGTTAGGTTAGGTT |
| Marker1077_61 | GGAAGAGATTAAAGAGAAGAGAGGAGCGAAGGAGGGAGACACTCATCCAC**[T/C]**CATTCACCTCCTCATCTAGCCATCAATCTATCCGATCCCACAACCTATCC |
| Marker1077_126 | TCTAGCCATCAATCTATCCGATCCCACAACCTATCCATCTACTCATCTAT**[C/T]**GATCCACCTCATCCTCCCTTCCCTTCCTTATCCGCTTATACGTCCCTTTC |
| Marker1077_72 | AAGAGAAGAGAGGAGCGAAGGAGGGAGACACTCATCCACTCATTCACCTC**[C/T]**TCATCTAGCCATCAATCTATCCGATCCCACAACCTATCCATCTACTCATC |
| Marker1077_65 | GAGATTAAAGAGAAGAGAGGAGCGAAGGAGGGAGACACTCATCCACTCAT**[T/C]**CACCTCCTCATCTAGCCATCAATCTATCCGATCCCACAACCTATCCATCT |
| Marker1077_146 | ATCCCACAACCTATCCATCTACTCATCTATCGATCCACCTCATCCTCCCT**[T/C]**CCCTTCCTTATCCGCTTATACGTCCCTTTCGTCCTCCGTTCCTTCTCTTC |
| Marker1077_134 | TCAATCTATCCGATCCCACAACCTATCCATCTACTCATCTATCGATCCAC**[C/A]**TCATCCTCCCTTCCCTTCCTTATCCGCTTATACGTCCCTTTCGTCCTCCG |
| Marker1077_81 | GAGGAGCGAAGGAGGGAGACACTCATCCACTCATTCACCTCCTCATCTAG**[C/T]**CATCAATCTATCCGATCCCACAACCTATCCATCTACTCATCTATCGATCC |
| Unigene2052_All__14026_558_197 | ATGCAGTGAAGGATTCCTGGATTCACTGAAGTAAAGTGGTGCGGTGCAAG**[G/A]**AAATACTGTTGACTGCAGTATATTACAGTCAAGAATGTGGAAGAAGAACA |
| Marker1077_98 | GACACTCATCCACTCATTCACCTCCTCATCTAGCCATCAATCTATCCGAT**[C/T]**CCACAACCTATCCATCTACTCATCTATCGATCCACCTCATCCTCCCTTCC |
| Marker9677_156 | TTCCCCAAAAACGCAAATAAATAGGTCTTGACTGCTGACATACAGCGGAC**[C/A]**CACACAGCACGCACAACACACGCGGCTCGCTCCACGTCACGAATAATAAT |
| Marker2060_197 | GGTTAGCAAATGTGAATGTTTGTATGTTAGTCTTTTGCAGTTATGATTGA**[T/C]**AATGTTCAATACGTGTTCTGCTTTTTTTTTTCTTTCTTTTTTTTTCTTTC |
| ALF6-1__22575_510_57 | GTTGTATTATATGTCACTATATTGTGTTAGTAAAACTACATTATGTATAT**[T/A]**ACATGTTACGGTTATCCTAGCTGGAATTGTACAAACACACACACACACA |
| Unigene19157_All__1806_348_223 | TCTGACGGTCTGTGCATCGGGGATAGCGGCACTCCGTCAGCTGTCGGAGG**[C/A]**GGCGACTGCAACACCCCGGCCTCACATCATAACCTGCGGAACCACTCCTT |
| Marker30980_113 | ATTCTCTGCTATCCATATTTTTCATCTCTTCTCTTAATTCTTCGTTCCTC**[C/A]**TCATTTTCTTTATTGTATCTTCATTATCACTTATTTTCCTTCTTTTTCTT |
| Marker4976_311 | TGTGTGTGGGTGGGTGTCCGGCTATGTGTGCGCATAGATTGTGATTCTTC**[A/G]**CCGAGTACCTGTGTATGCTTGGGTGTGGGTACCTGTGAATGGGTGTGGAT |
| Marker66_168 | TATAGTCATCACGGCACGCAGTCATAGTACGACAGTCATCGAAATATGGA**[A/T]**CAGTCACAGCAGCTGCCACAACTCACAAGCATTGTGCCGCAGTCATCATA |
| Marker4976_524 | TCATACGTAAAATGTTTTAGGTCTTTTACATCACCTGGGTGTTTTACCCT**[T/G]**TAAACACTATGTACGCTTGTATGTATGTACAGCATGCACCGTGTGTGTAT |
| Marker17240_299 | CTTGCACATACACATCCCACTTTTTATACATGGCGGAGTCTTTTTTTAGG**[C/T]**CCGTTACCCGTAATCGAGGTCCGAGGGCAGGCGAGCGGGCAAAACGCCTC |
| Marker8591_231 | GAGGGTAGAGGTTTTCTGAAGTCTGCTGCACTCCCCTCTTCCCTCGCTCA**[C/T]**TTGCCTCATTATCTTGCATCGCCTCAAAGACATTGTAAACCCCTCATATT |
| Marker66_291 | TCGCTACGACAGACGATCATCACTCAGTCATTACAACACCCGTTCATTAT**[A/G]**ATGCAATCACTACAACACACGCTAACCACAACACGGCCACTACACTTCAG |
| Marker4976_523 | GTCATACGTAAAATGTTTTAGGTCTTTTACATCACCTGGGTGTTTTACCC**[T/G]**TTAAACACTATGTACGCTTGTATGTATGTACAGCATGCACCGTGTGTGTA |
| Marker10592_316 | CTCTCCGCTCTCCCGCTCTGACTGATTGGGCGCTGACTGTCACGTGTCTC**[G/A]**TTTCGTTTTTTTTTTTTTTTTTTTTGTCGATTTTCTCGGTTTTATTTACT |
| Marker4976_316 | GTGGGTGGGTGTCCGGCTATGTGTGCGCATAGATTGTGATTCTTCACCGA**[G/A]**TACCTGTGTATGCTTGGGTGTGGGTACCTGTGAATGGGTGTGGATAATCC |
| Marker4311_49 | TCGCGCGTTCGAGTATCTTGTACGCCGAAATTTCTCTCATATCAGAGG**[C/A]**CCAACGCTCCAATAATTGTTATATTTTAAAGAAGCGAAATAGAAAGTTAA |
| ALF6-1__22575_510_224 | GGCGTTCACAACACCGGATTTGCTGCGGGTGTTGGCTGCAAGGGAGAACA**[G/A]**AACGGGAAGTTAAGGCTAAAGGAAGTTTGTATTGTATTACTCCATCTTTC |
| Marker16977_128 | TTCATATGTTCTAAACTTTTAATTTGCGCAACAGTATTGTATGATAAAAT**[T/G]**TATTATTTTAGGTTTAAAGTTTCCTATTATTGTCAGCAACAATAAAACAT |
| Marker10592_344 | GGCGCTGACTGTCACGTGTCTCGTTTCGTTTTTTTTTTTTTTTTTTTTGT**[C/G]**GATTTTCTCGGTTTTATTTACTTTCATTGTTTTATTTCAGTCTCCCCTAC |
| Marker11943_195 | TTTTCTTTGTCCACCTAAAAATCAGAAAATGTAGAATGTGCTGAGTGTGT**[T/C]**ATTTTCTGGTTATTTGTTATTGTGACTGTTGTTGTATGTATTACTTTCTA |
